# Reshoring Decisions for Adjusting Supply Chains in a Changing World: A Case Study from the Apparel Industry

**DOI:** 10.3390/ijerph18094873

**Published:** 2021-05-03

**Authors:** Pourya Pourhejazy, Alison Ashby

**Affiliations:** 1 Department of Industrial Engineering and Management, National Taipei University of Technology, Taipei 10608, Taiwan; pourya@ntut.edu.tw; 2 Exeter Business School, University of Exeter, Exeter EX4 4PU, UK

**Keywords:** decision analysis, reshoring, supply chain, competitive factors, sustainability

## Abstract

Global conditions for manufacturing are evolving rapidly and the myopic financial factors that once made overseas locations attractive for offshoring are now in favor of revising it. Besides, the COVID-19 pandemic has highlighted the need for restoring the previously offshored competencies. As a strategic decision, reshoring requires a balance of short- and long-term financial and non-financial considerations. This study extends the reshoring literature by exploring the underpinnings of the decision. For this purpose, the extended fuzzy Decision-Making Trial and Evaluation Laboratory (DEMATEL) is used to study the interrelationship among the decision criteria and explore the sequential effect of the prominent criteria on reshoring decisions. Data from the UK apparel industry is used as a baseline to provide insights for other industry situations. Findings are supportive of the supply process complexity as the prominent considerations with the highest potential impact on the financial criterion. Along with supply process complexity, environmental sustainability appears to have had the highest influence on cost-efficiency as the major driver of past offshoring decisions. Overall, the research findings provide insights for deeper analysis of the manufacturing location decisions for a globalized setting.

## 1. Introduction

Offshoring is the decision to locate production, supply, Research & Development (R&D), activities, and/or services to a foreign location outside the company’s home country [1]. The early 1990s to mid-2000s witnessed an exponential growth in the offshoring of manufacturing processes with key drivers being low-cost raw materials and labor that were available from developing countries [2], as well as improving operational efficiency [3]. These drivers were seen as a response to increasing competition, the progressive globalization of the economy caused by the lowering of trade barriers, and the emergence of new market players [4]. Additional factors pertinent to the location decision include tax rates, tariffs, and energy costs, and currency changes [5]; these factors offered substantial cost savings/advantages and were considered to be major reasons for offshoring’s growth [6].

Myopic offshoring decisions—the offshoring sins—include relocating the wrong activities, selecting the wrong vendors, putting poor contracts in place, lack of consideration of problems that may occur at the supplier, e.g., strikes, loss of control over the offshored activities, overlooking the hidden costs, and failing to plan an exit strategy [7]. These collected risks, issues, and implementation challenges are encouraging companies to bring part or all of production/services and supply bases back home or in closer proximity [5,8]; the reversion of a previous offshoring decision is typically framed as reshoring. The joint analysis of offshoring and reshoring is seen as a key component to better understanding long-term internationalization strategies and identifying when reshoring directly results from a failure of an offshoring strategy or represents the most appropriate strategic decision for a firm [9]. Understanding the way companies undertake the decision to reshore and implement it, and evaluate the result of that choice is of paramount importance [10]. Although the reversal of offshoring decisions is not a new phenomenon, the most recent instabilities in the international business environment, trade wars, and the pandemic have fueled the reshoring phenomenon; the exacerbated negative impact is making more firms revise their offshoring decisions to revive the core competency or switch to a new one, to stay competitive in a highly changing world.

Reshoring is not a mass phenomenon, nevertheless, it is a momentous topic with a range of policy and managerial implications that warrant academic research [11]. The imperative for academic research is increasingly recognized [10,12], but reshoring has been a practice-led phenomenon and as a result, the decision-making process is still under-researched. There is a need to understand what information is needed before and after making a reshoring decision and whether all this information is required before a decision can be made [13]. In addition to controversies surrounding the topic, the development of the field has also been affected by the limited availability of data, the unit of analysis often being at the product/component rather than the plant level, as well as limited research on the time dimension of offshoring/reshoring projects [8]. The decision of where to internationally locate a firm’s activities should be conceptualized under an incremental perspective, which dynamically evolves over time [12].

The reshoring literature has to date reported positive performance effects in terms of cost, quality, flexibility, and lead time, and there is a strong recognition of the relationship between the strategic motivation for reshoring and the expected performance outcomes [14]. While location decisions may appear primarily to be operative in nature, their potential strategic consequences are significant; the loss of strategic capabilities as a negative impact of all in offshoring has motivated researchers to conceptualize reshoring as a key means to restore long-term competitiveness [9,15]. The location decision is increasingly complex and time-sensitive [16] and multiple ownership and control structures can be considered [15]. Well-informed reshoring decisions considering a set of tangible and intangible supply chain-related criteria, which is in favor of reshoring the right portion of the offshored capacity, represent the right-shoring decisions and impact all businesses across the world [17,18].

The right-shoring concept is understudied and requires further investigation in academic literature. Of the existing literature, the majority are conceptual [19]; no studies have explored the interrelationship amongst decision criteria to understand the underpinnings of reshoring decisions. Financial criteria have dominated location decisions when they require a balance of short-term and long-term considerations. Like other strategic initiatives that evaluate different criteria, and their long-term implications, reshoring and offshoring decisions should be made using multiple and interrelated criteria, not just cost. This study aims to extend the limited body of reshoring literature to explore supply chain operational, competitive, and sustainability criteria within a broader organizational and inter-organizational context. Given the prevalence of offshoring within labor-intensive industries [20], a case from the UK Apparel industry is used to provide insights for more general industry situations. The results offer insights for industry practitioners and decision-makers, and directions for more dynamic and deeper research into modeling and managing reshoring decisions.

The remainder of this article begins with a review of the literature, followed by identifying the criteria pertinent to reshoring decisions in Section 2. Data collection, processing, and analysis are provided in Section 3. Finally, the major remarks and suggestions for future research directions conclude this research in Section 4.

## 2. Literature Review

### 2.1. Background

The location decision is a dominant topic in international business and supply chain management research and practice [10]; while offshoring and outsourcing originated in the manufacturing sector, they have also been implemented extensively in the service sector e.g., the movement of call centers to overseas locations [4]. Offshoring and outsourcing have been among the most widespread strategies adopted by Western firms since the early 1990s and have been used to maintain or foster competitive advantage [9,11]. Offshoring represents the relocation of production and other value chain activities to lower-cost countries [11] and it is distinguished from outsourcing by the employed ownership structure [21]. Offshoring equates to moving manufacturing out of the firm’s home country to an owned subsidiary, i.e., ownership and control are kept in-house, whereas in outsourcing the ownership and control are transferred to a third party [22]. While earlier research shows that bigger companies tended to be more active in offshoring than small or medium-sized firms, SMEs, there is no difference when it comes to reshoring [22,23]; current evidence suggests that companies of all sizes and from nearly all industries need to consider the configuration of value chains as a critical determinant of success [15].

Offshoring provides a potential path to price reductions and increased flexibility, allowing firms to convert fixed costs into variable expenses, and increase their economies of scope [24]. It often starts with the transactional and administration aspects of finance and human resources, but can extend to IT, supply, and support functions [4]. Overall, there are four distinctive managerial phases for offshoring, namely pre-assessment, evaluation of various alternatives, implementation, and post-evaluation. Offshoring alternatives include relocation to newly constructed plants or companies, e.g., foreign direct investment, relocation to an existing facility, or outsourcing to an overseas subcontractor who is not affiliated with the organization. As one of the possible decisions in the post-evaluation phase, reshoring refers to the reversal of a previous offshoring decision. 

Reshoring, as a cyclical process [13], is essentially a voluntary corporate strategy and location decision and it can be characterized as a revision of a previous offshoring project where production was either relocated to the company’s home country or a foreign country in the same region of the focal firm’s home country [8]. This revision of a previous offshoring decision can be further characterized as either a correction of previous managerial mistakes or a reaction to internal and/or external environment changes [25] and the emergence of new markets. The internal environment motivations are firm-specific, whereas external environment motivations relate to changing characteristics of the home/host countries [26]. Overall, potential reshoring strategies are (1) home reshoring, because of the failure of an earlier offshoring decision; (2) tactical reshoring which relates to short-term decisions based on the availability of resources and capabilities; (3) development reshoring if the firm aims to upgrade the proposed products [18].

The type of reshoring decision i.e., strategic choice or reaction to failure can impact the firm’s assessment of its capabilities as well as the aims of the reshoring decision i.e., strategic long-term vs. risk-mitigating short-term [10]. Reshoring does not necessarily imply relocating the activity to the country from where it was originally offshored, but could mean that it is moved to a facility in another country owned by the company [22]; it encapsulates near-shoring, i.e., relocation to closer proximity, back-shoring, i.e., return to the home country, and farther offshoring. Independent of who is performing the manufacturing activities [5], the terms reshoring and back-shoring are used interchangeably in the field; both have been referred to as the geographic relocation of a value-creating operation or process from a location abroad, back to the country of the focal firm, or re-integrated within the firm’s boundaries. A key common definitional feature is the reference to a strategic change [16]. While there is industry evidence of growth in reshoring, there has been limited research into the criteria for revising the previously made offshoring decisions; in particular, non-financial aspects were predominantly neglected when deciding to offshore. The following section explores the tangible and intangible criteria pertinent to reshoring decisions.

### 2.2. Tangible and Intangible Criteria for Reshoring

Reshoring decisions recognize firm-specific criteria such as offshoring misjudgments, global competitive dynamics, variables related to both the host and home countries, and the physical and cultural distances within the supply chain [13]. Location-specific advantages include (1) direct opportunities for cost reduction; (2) availability of resources, proximity to customers, and other network nodes; (3) cultural, political, legal, economic, and infrastructure characteristics of the host country. Given the ‘dispersed manufacturing’ phenomenon in supply chains, particularly in Apparel companies [27], right-shoring decisions should account for supply chain criteria. Moving part or all activities to a firm’s home country or a closer proximity location can be related to three distinct supply chain-related aspects: operational, tactical, and strategic. The operational aspect includes financial and tangible considerations which directly impact cost-efficiency. The tactical aspect comprises less tangible considerations that indirectly influence the company strategy and have a long-term influence on the cost-efficiency of the company. Finally, the strategic aspect determines the competitiveness of the company [28] and includes intangible considerations with a relatively small direct influence on its cost-efficiency.

#### 2.2.1. Operational and Tactical Aspects

Supply chain operational decisions in the manufacturing industry are traditionally evaluated in terms of cost, time, flexibility, quality, and innovation [29,30]; cost is recognized as the key driver for offshoring, while reshoring can be motivated by multiple additional considerations, including quality, flexibility, and the changing world [14].

Cost-oriented reshoring responds to the unforeseen costs of offshoring [14] including the closing labor cost gap and rising costs of transport and inventories [11]. Current overseas destinations for low-cost offshoring are experiencing increased pressure for wealth and welfare, and this translates into higher salaries and a closing of the gap in wage differences between developed and developing countries [22]. Higher oil prices, increased transport costs, and global supply risk in the current economic climate [31] have also exacerbated the cost-efficiency of offshoring.

Time-related reshoring motivations are particularly relevant in the case of geographically distant countries and mainly concern transport and logistics [9]. In-depth offshoring/reshoring decision-making needs to simultaneously consider both the facility location and subcontractor/supplier selection criteria [32]. Flexibility-oriented reshoring is more focused on the growing demand for product customization, lower volumes, and wider variety, with additional factors such as incentives, a shrinking market, and resolving an incorrect decision [23]. There can also be firm and product-specific characteristics, which influence location choice, particularly concerning product features [8].

Quality- and innovation-oriented reshoring relates to the upgrading of products, value-added operations, and the need for collaboration with the R&D and marketing functions, which is easier to achieve when all activities are in the same location. Current literature suggests that there is an intimate relationship between reshoring and various forms of technological innovations and developments in manufacturing, such as intelligent supply chains [33]. Knowledge and competence factors play an important role in the ongoing reshoring trend, especially when technology is the main driver [16]. Companies are reshoring part or all of production/service activities because it offers opportunities for new product development, enables better product quality, facilitates customization and delivery, and can even enable cost leadership [15].

Although costs are still a key consideration, it is firms with the capabilities of absorptive capacity, change readiness, flexibility, and agility who will be better positioned to reshore a function with fewer transaction costs [14]. Key benefits of reshoring include improved quality, reduced risk of intellectual property, improved flexibility, improved speed and simplicity, greater supply chain visibility, and an increased emphasis on and response to sustainability responsibilities [2].

#### 2.2.2. Strategic Aspect

The primary reasons for offshoring and reshoring are similar as firms will make sourcing decisions that best position them to establish, or improve, a strategic fit, i.e., cost-effectiveness, responsiveness, or differentiation of the products/services. Reshoring decisions should focus more on the long-term implications rather than reacting to trigger events [34], while the strategic disadvantages of offshoring include loss of tacit knowledge, loss of confidential data and violation of intellectual property [8], reduced innovation through physical and sometimes cultural distance, longer, more complex supply chains, higher transport costs, long lead times, limited flexibility, quality management difficulties and sustainability issues [2].

From a demand viewpoint, a higher willingness by customers to pay for reshored products can motivate companies to relocate production activities, as it increases customer perceived value; the positive “*Made In*” effect particularly encourages firms to relocate the entire value chain back to the home country [9]. From a supply perspective, proximity, both geographic and cultural, facilitates the development and maintenance of a close relationship between a buyer and a supplier; the extending of the supply process complexity through offshoring made such relationships more difficult to maintain [5]. Besides, any investment into supplier training can be lost when the relationship ends and the supplier may engage in opportunistic behavior through the sharing of information and demotivation of internal staff [8]. There may also be employee demotivation and/or a lack of specific capabilities at the local level, which results in poor customer service [4].

Finally, the risks and network externalities associated with offshoring, and the differences between overseas locations such as culture and language are less quantifiable than the cost advantages [5]; these considerations often highlight the key disadvantages or ‘hidden costs’ of offshoring. Offshoring also has significant environmental and social implications due to differences in country practices and standards. Reshoring can be motivated by a desire to reduce the risk of such environmental and social issues [2,35], and emotional reshoring further reflects location decisions that are made as a result of doing what is believed to be right [13]. The reshoring decision increasingly results from a greater emphasis on sustainability, with closer proximity to the home company enabling greater control over the environmental and social impact of manufacturing processes and a reduced environmental impact due to reduced transport [5,35].

Table 1 summarizes the range of decision criteria for reshoring, as well as the perceived benefits derived from each.

## 3. Data Collection and Processing

### 3.1. Data Collection

Data collection is conducted in two phases. First, the criteria identified through the literature review are screened by the experts to ensure the completeness and relevance of the criteria, and as a result, the Innovation and Inventory management criteria were unanimously identified as being poorly relevant in the context of the apparel industry. Following this phase, three established UK-based apparel manufacturers were identified and a file comprising factors definitions and a thirteen-by-thirteen matrix sent to them to complete. The chosen companies are all owner-managed Small and Medium-sized Enterprises (SMEs) that operate global supply chains, but they also aim to position some of their activities in the UK or a closer proximity location and thereby make both offshoring and reshoring decisions. The participants were two company owners and one supply chain manager, and they are hereafter denoted as our experts from the Apparel Industry. Their completion of the matrix addressed the following question:

When assessing “reshoring” and “offshoring” decisions, how much does each factor in the rows influence the factors at each column?

Five linguistic terms were established to define the extent of the interrelationship between two criteria: no influence (N), very low influence (VL), low influence (L), high influence (H), and very high influence (VH). The outcomes shown in Table 2 are used for data processing in the next sub-section.

### 3.2. Data Processing

The DEMATEL method [47] is used to explore the interrelationship between the decision criteria and identify the key considerations pertinent to reshoring decisions to help understand the system underpinnings of the decision [48]. DEMATEL has no cut-off value for the number of respondents [49]; hence, it can offer reliable outcomes using inputs from a limited number of experts [50]. The subjectivity of experts’ opinions makes the DEMATEL outcomes susceptible to uncertainty, imprecision, and vagueness, because the information may be filtered through the responders’ perception despite using realistic and unbiased data [51]. Fuzzy set theory [52] is used to lessen these deficiencies [53].

The computational process of Fuzzy DEMATEL begins with separately processing *n* experts’ opinions on the relationship between criterion *i* and *j*. For this purpose, five linguistic terms are defined to generate crisp values considering fuzzy set theory: N, VL, L, H, and VH are replaced by corresponding fuzzy triplets of (0, 0, 0.25), (0, 0.25, 0.5), (0.25, 0.5, 0.75), (0.5, 0.75, 1.0), and (0.75, 1.0, 1.0), respectively. Each triplet specifies an interval scale consisting of lower-bound ($fl_{ij}^{n}$), middle-point ($fm_{ij}^{n}$), and upper-bound ($fu_{ij}^{n}$) parameters. To prepare the direct-relation matrix (DRM), the values of the fuzzy numbers should be first transformed to the crisp equivalents. For this purpose, the interval values are first normalized by dividing their difference from the minimum value by the range of the fuzzy triplet. Then, the three-parameter values, $xl_{ij}^{n},xm_{ij}^{n},xu_{ij}^{n}$, are transformed to a double-parameter $xls_{ij}^{n},xus_{ij}^{n}$, first, and to the crisp equivalent ($d_{ij}^{n}$), next. Finally, the aggregated DRM should be obtained as the weighted average of the crisp values.

The next phase consists of normalizing DRM, i.e., dividing all the values by the greatest column-and-row summation value of the DRM table, resulting in the normalized direct-relation matrix (*NDRM*). To calculate the Total Relationship Matrix (TRM), *NDRM* will be multiplied by the reverse of the difference from the identity matrix, $TRM=NDRM{\left(1-NDRM\right)}^{-1}$.

The final phase consists of cause-effect, prominence, and sequential effect analysis [44]. For this purpose, the sum of row *i* in TRM determines the total influence the criterion *i* decision criteria dispatches, $D_{i}$, and the sum of column *i* shows the overall influence criterion *i* receives, $R_{i}$. On this basis, the prominence value of criterion *i* is $D_{i}+R_{i}$, which estimates the extent of its participation in the reshoring decision; the higher prominence values are interpreted as a criterion’s significance in the decision-making process. Besides, the role of criterion *i* in the decision environment is determined by the net-causation value, $D_{i}-R_{i}$. A criterion associated with a net-causation greater and smaller than zero refers to an influencer criterion and a criterion that is significantly influenced by others, respectively. Finally, the extent to which one criterion can sequentially impact the other criteria will be analyzed considering two measures; (1) the prospective influence based on the TRM matrix; and (2) the directional magnitude of the influence, which hinges on the current state of the prominent criteria. Based on this concept, a desirable or undesirable criterion is not likely to cause a significant change if it is connected with another criterion through a minor total relationship. Otherwise, significant interrelationships amongst two criteria can improve or worsen the sequential estimate of each other if the current state of the cause criterion is either desirable or undesirable, respectively.

### 3.3. Results Analysis

Given that shoring decisions are made for the long-term and the implementation is capital-intensive, it is of paramount significance to consider the sequential effect of the decisive criteria along with the temporal measures. That is, the decision-makers should consider the possible changes in the future state of the market environment as a result of the present changes in the indicators associated with the decision criteria. To analyze the total relationship matrix in Table 3, significant influence values are identified considering the mean plus 1.5 standard deviation of the total relations, as suggested by previous studies [44,51]. It is observed that the power of suppliers, product quality management, environmental sustainability, social responsibility, as well as uncertainty criteria have the highest potential to increase the supply process complexity. Notably, this influence appeared to be reciprocal. Besides, supply process complexity and the issues pertinent to environmental sustainability impose the highest influence on cost-efficiency, hence, should be observed when revising the offshoring decisions. In this situation, if the current state of the above criteria is perceived as underperforming, they likely impose a negative influence on the financial performance in the long term, hence, it may be logical to avoid complete back-shoring of the manufacturing activities.

We now analyze the prominence and net-causation values obtained from DEMATEL analysis with the results summarized in Table 4. Supply process complexity is recognized as the criterion with the highest prominence, meaning that this aspect should receive the highest attention to ensure “right-shoring”. The complexity of the supply process depends on the type of relationship with the suppliers, i.e., long-term vs. transactional relationships, and relationship closeness. Besides, reputation for integrity and trust are among the supplier relationship-related factors that may influence a reshoring decision.

As expected, cost-efficiency, supply chain speed, and the impact of the cultural differences are influenced significantly more than they dispatch influence on the other criteria considering the relatively large negative net-causation values. Increased labor costs in developing countries due to higher salaries, along with increased energy costs, rising logistics and freight costs have a significant impact on the cost-efficiency of the supply chain and production of products. On the other hand, timeliness of supply, on-time delivery, shorter lead times, and reduced risk of delays favor the near-shoring decisions, if the company aims to provide responsiveness in an attempt to adjust its strategic fit. From a brand perspective, if the customers’ perceived value is in the *Made-In*, the label of home-produced goods may or may not be desirable. That is, high-quality perceptions with specific countries e.g., Italy for fashion products improve customer brand perception; these factors may be significantly changing in the long run as a result of changes in the status of the other criteria introduced in the study. Finally, it is observed that environmental sustainability is the criterion with the greatest net influence. Considering the strategic nature of the decision, firms benefit from applying long-term perspectives, particularly because the stakeholders are increasingly willing to accept lower profit margins, emphasizing the trust and share in the firm’s sustainability vision.

### 3.4. Managerial Implications

Offshoring has been prevalent in industries where the cost savings are substantial following quota removal, with low-cost raw materials and labor from developing countries being the key initial drivers [20]. The global conditions have evolved rapidly within the past decade and the myopic financial factors that once made overseas locations attractive for offshoring are now in favor of reshoring of activities that have been offshored. However, reshoring comes with difficulties and challenges; the loss of control over processes and activities that may have been resulted from offshoring can make such decisions irreversible [6]. The process of offshoring has been so intense in certain industries that some manufacturing stages and skills have almost disappeared in the home countries [2]. This reflects the challenge of restoring the previously offshored product and process competencies and being able to respond to the loss of both tangible and tacit skills and knowledge in the home country to deal with unforeseen situations, like the recent pandemic. These challenges are industry-, product-, and country-specific, hence, case studies are required to examine these barriers.

Although the primary objective of reshoring may be the focus on core competencies/capabilities, it may also result in service improvement and standardization of processes. Revising previously made offshoring decisions should account for the influence of supply chain operational, tactical, and strategic considerations on the future financial aspect of the updated location decisions. As a prime example, the resulting sustainability and supply process simplicity after right-shoring can bring about sequential improvement to the cost-efficiency of the operations in the long term. Besides, right-shoring decisions may be required for reaching net-zero emission target for major emitting countries. Therefore, plausibly strict environmental regulations in the future may further increase the supply process complexity. In this situation, right-shoring amounts to establishing distributed production capacity.

A notable implementation factor to reshoring is the potential high investments in plant and equipment that would be needed to be made when a company plans to manufacture internally. While firms may be increasingly willing to restore offshored activities, there can also be significant disruptions, extensive impacts on management time, issues of quality, and consistency while the reshoring decision is implemented. A firm’s reshoring readiness, i.e., the extent to which they have considered and prepared for such a change, therefore, requires the criteria introduced in this study and their sequential effect. This is an extension to the earlier studies, like [16], that called for considering intangible, technology, and supplier/partner resources to improve reshoring readiness.

## 4. Conclusions

Reshoring consists of the decision to take back an activity, which had formally transferred to a foreign country, to the focal company, or a third party located elsewhere. From a global perspective, reshoring can be justified only if it contributes to both global and local sustainability; therefore, it is essential to consider the triple-bottom-line considerations collectively when approaching reshoring/offshoring decisions. Given the recent trend—notably, the pandemic—firms are reshoring activities to gain expertise internally where the developed competencies can be significantly more important than other location factors such as cost or market proximity. In this situation, there is a recognized need to understand the decision-making process for bringing activities back to the focal firm [10,13,54], hence, the *why* of reshoring was at the center of this paper’s focus. We found that supply process complexity plays a major role in reshoring decisions. Besides, social and environmental sustainability appeared to have a long-term impact on the cost-efficiency of the post-reshoring decisions. Overall, this study urges companies to consider the supply chain operational, competitive, and sustainability factors should they revisit the offshoring decision.

This study sets the foundation for deeper analysis of reshoring decisions considering country- and industry-specific situations. Quantification of the introduced intangible criteria can contribute greatly to the development of multi-objective optimization studies in the context of location planning and analysis. Besides, the interrelationship between the intangible and cost-efficiency factors can be further investigated in analytic studies, i.e., using big data. Given the ambiguity and dynamics involved in the reshoring decision, the next suggestion for future research is investigations that account for the instability of the decision environment. The Concept of Stratification [55] can be adapted to address the mentioned complexities. In so doing, the environment in which the relocation decisions occur can be characterized by a set of state variables, each of which influences the sustainability measures. Of the big set of state variables, some are more influential than the rest. Besides, the state variables are ambiguous and hard to be precisely forecasted. In this situation, the best decision is the one that is likely to work successfully under different circumstances. From an operational standpoint, achieving the highest environmental efficiency may be too costly at the current state; to address this point, the target set incremental enlargement concept, as another key Concept of Stratification’s strength, helps in integrating a proactive view towards sustainability aspects in reshoring within multi-criteria decision-making context. Finally, despite using managerial insights from a company where the reshoring decision is imperative, this study is limited in that it uses inputs from one industry and considers a small number of respondents. Comparative analyses across industries can be conducted in future research to determine how reshoring considerations, criteria importance, and criteria interrelationships may be contingent upon the industrial context.

## Figures and Tables

**Table 1:** Criteria for reshoring decisions.

Aspect	Criteria	Perceived Benefits	Supporting References
Operational	Cost-efficiency	Shrinking labor costs between developed and developing countries; Rising logistics costs will have a significant impact on goods being transported from overseas suppliers.	[11,22,31,36]
	Quality Management	Poor quality is associated with offshored manufactured products; Product and process quality differences between overseas locations; Opportunity to achieve higher quality through better control and inspection; Customer perceived value of the product.	[6,14,31]
	Supply chain speed	Timeliness of supply; On-time delivery; Shorter lead times; Reduced risk of delays	[12,36,37,38]
	Operational flexibility	Greater flexibility to demand changes; Better/quicker responses to market trends and new product development; Improved time to market for new products.	[6,39,40]
	Innovation	Co-location of R&D, engineering, and production; Better access to the latest technologies; Co-creation and customization opportunities with customers.	[10,11,14,22]
Tactical	Inventory Management	Global supply chains hold inventory to mitigate delays and market changes, while the growth in lean and agile supply chain strategies require minimal- or zero-inventory.	[38]
	Supply process complexity	Simpler supply chains improve information sharing. In addition to relationship closeness with the suppliers and the extent of control and ownership stake that facilitates the supply process, ease of import/export considering Incoterms can also be influential.	[35,41,42]
	Supply chain visibility	Geographic distances present challenges in supplier monitoring and management; Closer proximity of suppliers can improve the visibility of processes and practices and better supply chain coordination.	[38,39]
	Supply and demand uncertainty	Uncertainty and volatility in the market; Institutional and regulatory changes; Variable exchange rates; Supply chain disruption risks.	[2,11,41,43]
Strategic	Access to skills & knowledge	Lack of workers with specific/required skills in overseas locations; Utilization of new technologies and automation; Improved knowledge sharing.	[10,11,14,22]
	Brand image and the company’s reputation	The brand perceptions with specific countries improve customer brand perception; this factor may influence the managers’ reshoring decision.	[9]
	Power of supplier	The fewer the suppliers/more a firm depends upon a supplier, the more power a supplier has. Many suppliers/low switching costs mean a firm can keep costs lower and increase profits. In this situation, revising reshoring decisions may be easier.	[28,44]
	Cultural differences impact	Language barriers and cultural distances impact the quality and frequency of supplier communication; Closer proximity can improve communication and enable the development of shared norms and values.	[35,45,46]
	Environmental sustainability	Opportunity for lowered carbon emissions and cleaner energy, more environmentally responsible processes, and better waste management/prevention	[40,43]
	Social responsibility	Job creation and increased employment; Economic development in the local region; More opportunity to address workers’ rights, fair wages, and working conditions.	[35,40]

**Table 2:** Criteria relation data obtained from the experts.

DRM-1	C1	C2	C3	C4	C5	C6	C7	C8	C9	C10	C11	C12	C13
C1	-	L	L	L	VL	H	H	H	H	H	L	L	H
C2	H	-	H	H	L	L	VH	H	H	H	H	H	H
C3	H	N	-	H	VL	H	H	H	H	H	L	H	H
C4	H	L	H	-	H	VH	H	H	H	H	L	H	H
C5	L	H	H	H	-	H	H	VH	VH	VH	L	H	H
C6	VH	VH	VH	VH	VH	-	VH	VH	VH	VH	L	VH	VH
C7	VH	VH	VH	H	H	VH	-	H	H	VH	H	L	VH
C8	VH	VH	VH	VH	VH	VH	H	-	H	VH	H	VH	VH
C9	VH	VH	VH	VH	VH	VH	H	H	-	VH	H	VH	VH
C10	H	H	H	H	VH	VH	H	VH	VH	-	L	VH	VH
C11	L	L	VL	VL	H	L	H	H	H	L	-	L	L
C12	H	H	H	H	H	H	L	L	L	L	L	-	L
C13	H	VH	VH	VH	VH	VH	VH	VH	VH	VH	L	H	-
**DRM-2**	C1	C2	C3	C4	C5	C6	C7	C8	C9	C10	C11	C12	C13
C1	-	H	VH	H	H	VH	VH	H	H	H	L	H	H
C2	H	-	H	L	L	L	VH	L	L	H	VH	L	H
C3	VH	H	-	VH	L	H	H	H	H	H	L	H	VH
C4	H	L	VH	-	L	H	H	L	L	H	L	L	VH
C5	H	L	L	L	-	VH	VH	VH	VH	VH	H	H	VH
C6	VH	L	H	H	VH	-	H	VH	VH	H	VH	VH	VH
C7	VH	VH	H	H	VH	H	-	L	L	VH	VH	L	VH
C8	H	L	H	L	VH	VH	L	-	H	H	VH	VH	VH
C9	H	L	H	L	VH	VH	L	H	-	VH	VH	L	H
C10	H	H	H	H	VH	H	VH	H	VH	-	L	L	H
C11	L	VH	L	L	H	VH	VH	VH	VH	L	-	H	H
C12	H	L	H	L	H	VH	L	VH	L	L	H	-	VH
C13	H	H	VH	VH	VH	VH	VH	VH	H	H	H	VH	-
**DRM-3**	C1	C2	C3	C4	C5	C6	C7	C8	C9	C10	C11	C12	C13
C1	-	L	H	L	L	VH	VH	VH	VH	L	L	L	L
C2	L	-	H	L	H	H	H	VL	VH	L	VL	L	VL
C3	H	H	-	L	N	L	L	H	H	L	L	L	L
C4	L	L	L	-	L	VH	VH	L	L	L	L	H	L
C5	L	H	N	L	-	H	H	VH	VH	N	VL	L	H
C6	VH	H	L	VH	H	-	H	H	H	H	H	L	L
C7	VH	H	L	VH	H	H	-	L	L	H	H	L	L
C8	VH	VL	H	L	VH	H	L	-	H	H	H	L	L
C9	VH	VH	H	L	VH	H	L	H	-	VH	L	L	H
C10	L	L	L	L	N	H	H	H	VH	-	L	H	H
C11	L	VL	L	L	VL	H	H	H	L	L	-	L	L
C12	L	VL	L	H	L	L	L	L	L	H	L	-	L
C13	L	VL	L	L	H	L	L	L	H	H	L	L	-

C1: Cost-efficiency, C2: Access to skills & knowledge, C3: Supply chain speed, C4: Supply chain flexibility/responsiveness, C5: Supply chain visibility, C6: Supply process complexity, C7: Quality management, C8: Social responsibility, C9: Environmental sustainability, C10: Operational and market uncertainty, C11: Brand image and the company’s reputation, C12: Cultural differences impact, C13: Power of suppliers.

**Table 3:** Influence analysis in the total relationship matrix (significant values in **bold**).

TRM	C1	C2	C3	C4	C5	C6	C7	C8	C9	C10	C11	C12	C13
C1	0.3703	0.3741	0.4121	0.3900	0.3962	0.4748	0.4489	0.4412	0.4472	0.4215	0.3577	0.3850	0.4259
C2	0.4101	0.3024	0.3926	0.3710	0.3827	0.4329	0.4296	0.3940	0.4201	0.4011	0.3539	0.3662	0.3988
C3	0.4186	0.3443	0.3210	0.3801	0.3511	0.4278	0.4034	0.4088	0.4142	0.3961	0.3356	0.3690	0.4071
C4	0.4155	0.3539	0.3977	0.3223	0.3880	0.4586	0.4278	0.4063	0.4119	0.4062	0.3443	0.3784	0.4177
C5	0.4352	0.3908	0.3954	0.3998	0.3611	0.4804	0.4531	0.4659	0.4722	0.4327	0.3674	0.4021	0.4506
C6	**0.5270**	0.4479	0.4770	0.4799	0.4957	0.4692	**0.5131**	**0.5183**	**0.5253**	**0.5027**	0.4362	0.4683	**0.5087**
C7	0.4931	0.4327	0.4456	0.4429	0.4563	**0.5076**	0.4070	0.4586	0.4658	0.4764	0.4146	0.4112	0.4754
C8	0.4937	0.4114	0.4586	0.4360	0.4774	**0.5220**	0.4670	0.4123	0.4864	0.4767	0.4206	0.4453	0.4828
C9	**0.5008**	0.4381	0.4654	0.4424	0.4839	**0.5294**	0.4746	0.4868	0.4263	0.4972	0.4197	0.4381	0.4898
C10	0.4529	0.4000	0.4260	0.4167	0.4312	0.4919	0.4642	0.4633	0.4831	0.3821	0.3761	0.4186	0.4613
C11	0.3771	0.3397	0.3471	0.3394	0.3663	0.4188	0.4024	0.4017	0.4001	0.3687	0.2773	0.3491	0.3795
C12	0.3897	0.3315	0.3672	0.3599	0.3710	0.4188	0.3759	0.3887	0.3803	0.3745	0.3299	0.2953	0.3861
C13	0.4696	0.4145	0.4548	0.4457	0.4667	**0.5103**	0.4819	0.4809	0.4875	0.4728	0.3968	0.4341	0.4040

C1: Cost-efficiency, C2: Access to skills & knowledge, C3: Supply chain speed, C4: Supply chain flexibility/responsiveness, C5: Supply chain visibility, C6: Supply process complexity, C7: Quality management, C8: Social responsibility, C9: Environmental sustainability, C10: Operational and market uncertainty, C11: Brand image and the company’s reputation, C12: Cultural differences impact, C13: Power of suppliers.

**Table 4:** Results analysis of the reshoring decisions.

Identifier	Criteria	Influence		Prominence	Net-Causation
		Dispatched	Received		
C1	Cost-efficiency	5.3450	5.7537	11.0986	−0.4087
C2	Access to skills & knowledge	5.0554	4.9815	10.0368	0.0739
C3	Supply chain speed	4.9773	5.3604	10.3377	−0.3832
C4	Supply chain flexibility responsiveness	5.1286	5.2262	10.3548	−0.0975
C5	Supply chain visibility	5.5067	5.4277	10.9344	0.0790
C6	Supply process complexity	6.3691	6.1424	12.5116	0.2267
C7	Quality management	5.8873	5.7489	11.6361	0.1384
C8	Social responsibility	5.9903	5.7269	11.7172	0.2635
C9	Environmental sustainability	6.0924	5.8204	11.9128	0.2720
C10	Operational and market uncertainty	5.6674	5.6087	11.2761	0.0588
C11	Brand image/company’s reputation	4.7671	4.8300	9.5971	−0.0629
C12	Cultural differences impact	4.7688	5.1607	9.9295	−0.3919
C13	Power of suppliers	5.9196	5.6876	11.6072	0.2319

## Data Availability

Data is contained within the article.
